# Results of feasibility and safety of randomised controlled trial of a musculoskeletal exercise intervention versus usual care for children with haemophilia

**DOI:** 10.1111/hae.14026

**Published:** 2020-05-06

**Authors:** Melanie Bladen, Liz Carroll, Charlene Dodd, Wendy Drechsler, Ferhana Hashem, Vishal Patel, Tracy Pellatt‐Higgins, Eirini Saloniki, David Stephensen

**Affiliations:** ^1^ Great Ormond Street Hospital for Children Heamophilia Centre London UK; ^2^ The Haemophilia Society London UK; ^3^ Faculty of Life Sciences & Medicine King College London London UK; ^4^ Centre for Health Service Studies University of Kent Canterbury UK; ^5^ Barts Health NHS Trust Haemophilia Centre London UK; ^6^ Kent Thrombosis and Haemophilia Centre Kent and Canterbury Hospital Canterbury UK

**Keywords:** exercise, haemophilia, paediatrics, physical function, physiotherapy

Dear Editor

Evidence indicates that despite prophylaxis, boys with severe and moderate haemophilia still bleed one to two times per year.^1^ It is well established that muscle weakness is associated with haemophilic arthropathy in adults^2^ and it is now becoming apparent that muscle strength may be reduced in children, prior to the onset of clinical arthropathy.^3^, ^4^

Therapeutic exercise is an important component of the management of other forms of arthropathy (eg osteoarthritis and rheumatoid arthritis),^5^ and it would appear logical that exercise would be effective for people with haemophilia. It is a commonly held view of some clinicians that increases in muscular strength might improve motor performance and cardiovascular fitness and limit exaggerated end‐range joint movement.^6^ It may also promote optimal transfer of weight‐bearing forces through joints, thereby minimizing muscle imbalance, synovial impingement and associated haemarthroses or synovitis.^6^ However, there is a lack of evidence to support these assumptions. A recent Cochrane Review evaluating the safety and effectiveness of exercise for people with haemophilia reported four randomized controlled studies of an exercise intervention in children with the condition and concluded the studies were of low or very low quality, due to small sample sizes and potential bias.^7^ Furthermore, no paediatric study compared a muscle strengthening intervention to a control group or intervention without muscle strengthening exercises. Additionally, pre‐adolescent and adolescent boys were included in the same study, and it is not known whether the groups were matched for pubertal status. In pre‐adolescent children, there is a linear relationship between strength, age and body size, but in young adolescent children, increases in strength are related to puberty rather than age which is likely to influence comparisons of strength and functional outcomes between children.^8^ Despite the apparent benefit, there is a lack of robust evidence to determine whether muscle strengthening exercise can improve or negatively affect outcomes for young children with haemophilia.

The purpose of this two‐centre feasibility randomized controlled trial (RCT) was to test the feasibility of an age‐appropriate physiotherapy intervention co‐designed by healthcare professionals and patients^9^ to improve muscle strength and physical function in pre‐adolescent boys with haemophilia. Favourable ethical approval was obtained from the Health Research Authority and London—Fulham Research Ethics Committee and (17/LO/2043).

The full protocol published elsewhere^9^ utilized a single‐blinded randomized approach. Following screening and informed consent, participants were randomly allocated into one of two groups (Treatment Group 1 received a 12‐week exercise intervention; Treatment Group 2 received usual physiotherapy care for 12 weeks) on a ratio of 1:1. The intervention developed by expert clinicians and patients utilizing a modified Nominal Group Technique is a 24‐session, 12‐week programme designed as 2‐week progressive levels (intensity and or load) with no more than 10 exercises in each session. The intervention aims to master movement control and emphasizes body‐weight strength development. Pictures of the exercises and instructions for each phase were provided in 2‐week exercise diaries. Participants completed the same exercises and were asked to complete the exercises twice per week: once with the physiotherapist who visited the participant at home and once supervised by their parents/ guardian. Completion of exercises, along with treatment regimen, any adverse events and comments in relation to the exercise programme were recorded in the exercise diary.

Primary outcome was safety and adherence. Secondary outcomes were recruitment rate: lower limb maximum muscle strength; six‐minute timed walk (6MTW); timed up‐ and downstairs (TUDS)^10^; EQ‐5D‐Y; and costs relevant to the study. Safety, adherence, recruitment and follow‐up are described as percentages. Demographic and disease variable distributions were analysed for descriptive purposes and covariant analysis. Estimates of differences between treatment arms in the changes from baseline (adjusted for baseline) and 75% CI were calculated to estimate the potential effect of the intervention and provide estimates of variability to inform the sample size required for a full RCT, as well as to refine the outcome measures by evaluating their responsiveness to change. Unit costs were combined with the duration, number of sessions recorded and number of clinicians involved in the sessions to provide a pragmatic intervention cost for the usual care and intervention groups. Individual‐level resource use was combined with unit costs to calculate the total health services cost for each participant. Hospital outpatient and A&E visits were combined with the national average (unit cost) from national databases such as the Unit Costs of Health and Social Care 2018^11^ and the United Kingdom Department of Health and Social Care National Schedule of Reference Costs (2017‐2018).

Nine children participated in the feasibility study: seven were diagnosed with severe haemophilia A and two with severe haemophilia B. Five were randomly allocated to the exercise intervention and four to usual care. Mean age of the children was 9.77 years (SD 2.18); mean height, 1.40 m (0.17); and mean BMI, 18.65 kg/m^2^ (3.11). We recruited 75% (target > 50%) of participants who met the inclusion criteria from two study sites; all were willing to be randomized, and the follow‐up retention rate was 100%. Participants reported feeling ‘satisfied with the group they were allocated and understood the logic of randomisation and needing a control arm of the trial’. They did not feel any detrimental impact for not receiving the exercise programme by being offered the exercise programme at the end of the study, which they were informed about during the consent process. We conducted a single‐blinded approach with outcome data collected by a physiotherapist blinded to the participant allocation. Outcome data were collected by the same physiotherapist at both sites. Blinding was maintained throughout the study with participants and physiotherapists delivering the intervention encouraged to withhold the group allocation from the assessor during data collection. Eighty‐three (83) per cent (24/29) of exercises were completed by all participants at least twice per week during the 12‐week intervention (target = 75%). Adherence to the intervention was 100% (exercises performed at least twice per week) in the first ten weeks of the intervention and 62.5% during the final 2 weeks. The mean time taken to complete each exercise session was 56.00 ± 5.48 minutes. Two adverse events were reported during the study: one lower limb muscle bleed following a game of football at school for one of the participants, and a knee joint bleed following a fall at the park for a second participant. Both events were reviewed by the Research Monitoring Group and considered not directly attributable to the intervention. There were no serious adverse events or adverse reactions reported during the study.

Although the efficacy was not the aim of this study, muscle strength of ankle plantarflexors and knee extensors, distance walked in six‐minutes and time taken to ascend and descend 12 steps improved in children receiving the intervention compared with those who did not (Table 1 and Figure 1). However, we acknowledge these findings in such a small sample are underpowered. The total actual intervention cost was £802.12 (SD £66.62) per participant, ranging between £714.10 and £860.80.

**TABLE 1:** ANCOVA for muscle strength, function and quality of life—change from baseline, adjusted means and 75% confidence intervals

	Usual care group (n = 4)	Exercise group (n = 5)	Difference	75% confidence interval
Maximum isometric muscle strength (Nm/kg)				
Left ankle dorsiflexors	0.0066	0.0365	0.0299	−0.0265 to 0.0863
Right ankle dorsiflexors	0.0189	0.0083	−0.0106	−0.0527 to 0.0316
Left ankle plantarflexors	−0.0100	0.1193	0.1294	0.0125 to 0.2462
Right ankle plantarflexors	−0.0575	0.1845	0.2420	0.1565 to 0.3275
Left knee extensors	0.0132	0.3456	0.3324	0.1068 to 0.5580
Right knee extensors	−0.1553	0.2828	0.4381	0.2548 to 0.6213
Function and quality of life				
Six‐minute timed walk (m)	−7.92	53.3	61.2	12.5 to 110
Timed up‐ and downstairs (sec)	−0.706	−2.43	−1.73	−2.51 to −0.942
EQ‐5D‐Y VAS	−5.08	1.36	6.44	−7.57 to 20.4

Abbreviations: ANCOVA, analysis of covariance; Nm/kg, Newton‐metre/kilogram; m, metre; sec, econds; VAS, visual analogue scale.

**Figure 1:**
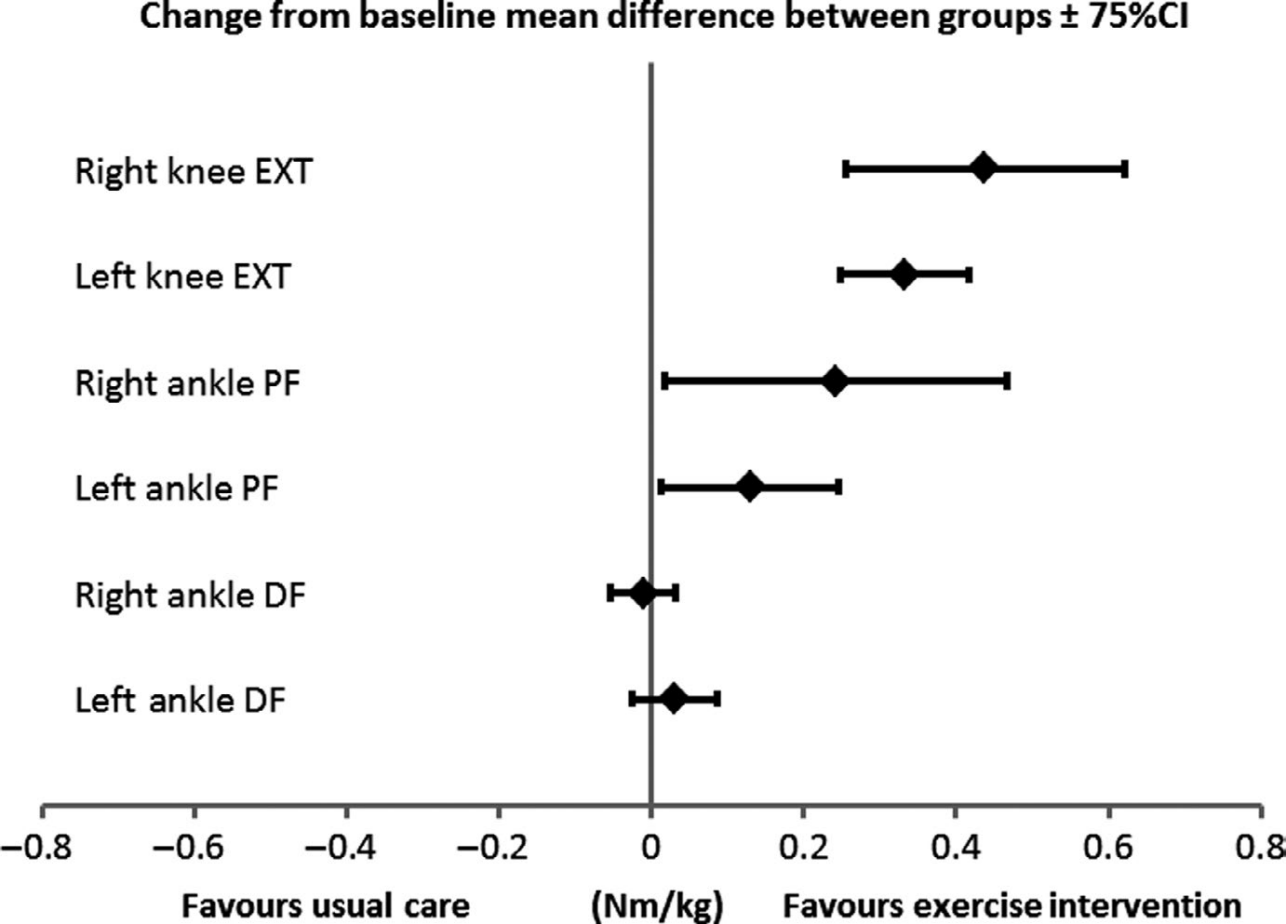
Group difference change from baseline for maximum muscle strength. CI = confidence interval; EXT = extensors; PF = plantarflexors; DF = dorsiflexors; Nm/kg = Newton‐metre/kilogram

In partnership with children with haemophilia, their families and healthcare professionals we have co‐produced a low‐cost intervention and study design that is safe and suitable to test efficacy on muscle strength, function and participation in physical activities in children with the condition. Our study strategies ensured high recruitment and retention. Random allocation was acceptable to those who participated, and we were able to successfully maintain a single‐blinded approach. We developed an intervention that had no adverse events and was acceptable to patients and physiotherapists that we plan to evaluate in a fully powered single‐blinded two‐arm pragmatic randomized controlled trial.

## DISCLOSURES

The authors stated that they had no interests which might be perceived as posing a conflict or bias.
